# Drinking to Cope or Coping to Drink? Behavioral Profiles of Stress Management and Alcohol Use Risk Among Medical Students: A Cross-Sectional Study

**DOI:** 10.3390/jcm15093218

**Published:** 2026-04-23

**Authors:** Lucretiu Radu, Madalina Aldea, Vlayko Vodenicharov, Teodor Nicolae Dinescu, Iulia Balutoiu, Ramona Constantina Vasile, Alexandra-Daniela Rotaru-Zavaleanu, Citto Iulian Taisescu, Andrei Gresita, Mihai Andrei Ruscu, Venera Cristina Dinescu

**Affiliations:** 1 Department of Hygiene, University of Medicine and Pharmacy of Craiova, 2-4 Petru Rares Str., 200349 Craiova, Romania; lucretiu.radu@umfcv.ro; 2 Department of Psychiatry, University of Medicine and Pharmacy of Craiova, 2-4 Petru Rares Str., 200349 Craiova, Romania; madalina.aldea@umfcv.ro; 3 Department of Epidemiology and Hygiene, Medical University Sofia, Boulevard “Akademik Ivan Evstratiev Geshov” 15, 1431 Sofia, Bulgaria; vlayko.vodenicharov@gmail.com; 4 University of Medicine and Pharmacy of Craiova, 2-4 Petru Rares Str., 200349 Craiova, Romania; teodor.dinescu@umfcv.ro; 5 Department of Psychology, University of Medicine and Pharmacy of Craiova, 2-4 Petru Rares Str., 200349 Craiova, Romania; 6 Department of Epidemiology, University of Medicine and Pharmacy of Craiova, 2-4 Petru Rares Str., 200349 Craiova, Romania; alexandra.rotaru@umfcv.ro (A.-D.R.-Z.); mihai.ruscu@umfcv.ro (M.A.R.); 7 Department of Physiology, University of Medicine and Pharmacy of Craiova, 2-4 Petru Rares Str., 200349 Craiova, Romania; citto.taisescu@umfcv.ro (C.I.T.); andrei.gresita@umfcv.ro (A.G.); 8 Department of Health Promotion and Occupational Medicine, University of Medicine and Pharmacy of Craiova, 2-4 Petru Rares Str., 200349 Craiova, Romania; venera.dinescu@umfcv.ro

**Keywords:** alcohol use, coping strategies, Brief COPE, AUDIT, medical students, cluster analysis, behavioral profiles, Romanian students, cross-sectional study

## Abstract

**Background/Objectives**: Alcohol misuse among medical students is commonly attributed to academic stress, yet the specific role of coping mechanisms in this relationship has received limited attention. We investigated whether substance use coping, rather than stress exposure itself, drives alcohol use risk in Romanian medical students, and whether distinct coping-based subgroups can be identified through cluster analysis. **Methods**: We conducted a cross-sectional survey among 244 medical students (mean age 21.95 ± 3.27 years; 67.2% female) at the University of Medicine and Pharmacy of Craiova, Romania. Alcohol use was measured with the AUDIT and coping strategies with the Brief COPE. Analyses included Mann–Whitney U tests, Spearman correlations, multiple linear and binary logistic regression, and k-means clustering. **Results**: At-risk drinking (AUDIT ≥ 8) was identified in 19.7% of participants. The tendency to use substances to cope with stress (substance use coping) was the strongest predictor of AUDIT scores in both linear regression (B = 2.090, *p* < 0.001, R^2^ = 0.513) and logistic regression (OR = 2.026, *p* < 0.001). Male sex independently predicted at-risk status (OR = 2.572, *p* = 0.025), while planning was protective in both models (B = −0.657, *p* = 0.005; OR = 0.691, *p* = 0.029). Humor also emerged as a significant risk factor (OR = 1.638, *p* = 0.005). K-means analysis (k = 5) revealed five coping profiles with significantly different AUDIT distributions (Kruskal–Wallis H = 47.26, *p* < 0.001). The Substance-Oriented cluster (13.1% of students) had a mean AUDIT of 12.66, compared with 3.00–4.13 in other clusters. **Conclusions**: In a subgroup of medical students, alcohol use appears integrated into the coping repertoire rather than merely being a consequence of stress. The identified coping profiles should be interpreted as prototypical configurations with overlapping boundaries rather than discrete categorical types, given the low silhouette coefficient (0.094) of the cluster solution. The strong predictive effect of substance use coping should be interpreted with the caveat that the Brief COPE Substance Use subscale and the AUDIT share content related to alcohol use behavior, which may inflate the observed association. These findings point to the need for coping-specific interventions. Planning skills training and a more nuanced understanding of humor’s role in drinking contexts may offer avenues for prevention. However, the logistic model’s sensitivity of 50.0% indicates that coping-based identification alone would miss approximately half of at-risk students, underscoring the need for further refinement before clinical application.

## 1. Introduction

Medical education exposes students to sustained cognitive demands, hierarchical training structures, and regular contact with human suffering [1,2]. Under these conditions, the range of available coping responses can narrow, and pharmacological alternatives, including alcohol, may become more appealing [3,4]. Alcohol is widely accessible, socially accepted in student culture, and provides short-term anxiolytic effects, which makes it a particularly convenient option.

The prevalence of hazardous drinking in medical students has been documented in numerous studies, with international estimates generally ranging from 10% to 25% and somewhat higher figures reported from Eastern European settings [5]. Recent data confirm that this pattern persists: Ay et al. (2025) reported a 13.5% prevalence of hazardous alcohol consumption (AUDIT ≥ 8) in a large Turkish university sample [6], while a Polish medical student cohort yielded a figure of 30.9% [7]. In the Romanian context specifically, depression and psychological distress among medical students have been systematically documented [8], but data on alcohol use and coping remain limited. Less clear, however, is whether alcohol use in this population represents a downstream effect of stress or whether it functions as a deliberate, if maladaptive, coping strategy. This distinction has practical implications: if alcohol serves a coping function, interventions focused solely on reducing stressors may be insufficient, and the coping mechanism itself needs to be addressed [9].

More broadly, the tendency to use alcohol as a coping mechanism may be rooted in underlying deficits in affect regulation, particularly difficulties in identifying and describing one’s own emotional states. Alexithymia, a construct encompassing impaired emotional awareness and externally oriented cognition, has been consistently associated with substance use disorders and maladaptive coping [10]. Individuals with alexithymic traits may turn to alcohol as a pharmacological substitute for cognitive–emotional regulation strategies they are unable to deploy, a pattern that may be particularly relevant in high-stress training environments where emotional demands are high but opportunities for reflective processing are limited. Recent neuroimaging evidence further suggests that the neurobiological substrates of alcohol use disorder overlap with brain regions implicated in affect regulation deficits, including the insula, anterior cingulate cortex, and prefrontal cortex [11].

The Brief COPE inventory is well suited for examining this question, as it includes a Substance Use subscale that operationalizes alcohol and drug use as strategies for managing problems. This allows a direct test of whether endorsing substance use as coping, rather than stress per se, constitutes the more proximal predictor of alcohol risk [12]. Most previous research on this topic in medical students has relied on variable-centered approaches (correlations, regressions), which describe average relationships across the sample [13]. Such designs can obscure the possibility that meaningfully different subgroups exist within the student population, groups with qualitatively distinct coping profiles that may require different types of intervention. Person-centered methods such as cluster analysis are designed to identify such subgroups [14].

Although humor is conventionally classified as an adaptive coping strategy, emerging evidence suggests that its relationship with substance use is more nuanced than this classification implies. Baldacci et al. (2025) recently demonstrated that self-defeating humor was a significant positive predictor of alcohol use as measured by the AUDIT, proposing that maladaptive humor styles may reinforce problematic drinking by serving as a dysfunctional coping mechanism in social contexts [15]. Similarly, Simione and Gnagnarella (2023) showed that humor coping moderates the relationship between avoidance strategies and perceived stress, indicating that its protective function may depend on the broader coping configuration in which it is embedded [16]. These findings suggest that treating humor as uniformly protective may obscure important context-dependent risk associations, a possibility that the present study examines directly.

The Romanian context is relevant for several reasons. Medical schools in Romania are characterized by competitive admission, traditional teaching hierarchies, and relatively limited student mental health infrastructure [8]. Romania also has one of the higher per-capita alcohol consumption levels in Europe, contributing to a cultural environment in which drinking is normalized [17]. Despite this, Romanian medical students have been largely absent from the international literature on alcohol use and coping [2].

We hypothesized that substance use coping would be the primary predictor of alcohol use risk in this sample, and that this risk would be concentrated in an identifiable subgroup rather than evenly distributed across the student body. We also aimed to examine sex differences in coping and alcohol use and to identify specific coping strategies with protective or risk-amplifying effects that could inform curricular interventions.

## 2. Materials and Methods

### 2.1. Study Design and Setting

A cross-sectional observational study was conducted among students enrolled at the University of Medicine and Pharmacy of Craiova, Romania. The study was conducted in accordance with the Declaration of Helsinki. Ethical approval was obtained from the Institutional Ethics Committee (approval no. 134/22 June 2022). All participants provided written informed consent prior to enrollment. Recruitment was conducted between January and February 2026 using a non-probability convenience sampling approach. The questionnaire was hosted on Google Forms (Google LLC, Mountain View, CA, USA) and disseminated through the university’s official student e-learning platform, as well as through faculty-approved student group communications (e.g., year-representative email lists and student organization channels). No classroom-based or face-to-face recruitment was employed. Potential participants accessed the survey link at their own convenience; participation was entirely voluntary, anonymous, and carried no academic incentives or penalties. A single reminder was sent approximately two weeks after the initial distribution. The study was promoted as an investigation into “coping strategies and lifestyle habits among medical students,” without specific reference to alcohol use in the recruitment material, in order to minimize selection bias related to personal drinking behavior.

### 2.2. Participants

A convenience sample of students was recruited from across all years of the undergraduate medicine program (years 1 through 6). Eligible students were those currently enrolled in the Romanian-language section of the Faculty of Medicine during the 2025–2026 academic year. The total eligible population comprised approximately 2000 students; 247 accessed the survey (estimated response rate ~10.3%). No stratified or quota-based sampling was applied; all enrolled students had equal opportunity to participate, and the sample composition across study years reflects self-selection rather than proportional representation. Inclusion criteria were (1) full-time enrollment in the Faculty of Medicine; (2) age ≥ 18 years; (3) provision of informed consent. Students who did not consent or whose sex was not recorded as male or female were excluded from the primary analytical sample. Of 247 respondents, 246 consented to participate and 244 provided complete data with binary sex classification (mean age 21.95 ± 3.27 years; range 19–41; 67.2% female [*n* = 164], 32.8% male [*n* = 80]).

### 2.3. Instruments

#### 2.3.1. Alcohol Use Disorders Identification Test (AUDIT)

Alcohol use was assessed using the AUDIT [18], a 10-item WHO-validated self-report instrument yielding a total score (theoretical range 0–40) and three subscale scores: AUDIT-C (Consumption, items 1–3; theoretical range 0–12), AUDIT-D (Dependence symptoms, items 4–6; theoretical range 0–12), and AUDIT-P (Alcohol-related Problems, items 7–10; theoretical range 0–16). Items 1–8 are scored 0–4 and items 9–10 are scored 0, 2, or 4 according to standard WHO guidelines [19]. Standard risk thresholds were applied: low risk (0–7), hazardous use (8–15), harmful use (16–19), and probable dependence (≥20).

#### 2.3.2. Brief COPE

Coping strategies were assessed using the Brief COPE [20], a 28-item inventory measuring 14 coping subscales on a 4-point Likert scale (1 = I haven’t been doing this at all; 4 = I’ve been doing this a lot). The 14 subscales are: Self-Blame, Positive Reframing, Substance Use, Emotional Support, Active Coping, Religion, Acceptance, Self-Distraction, Instrumental Support, Denial, Venting, Behavioral Disengagement, Planning, and Humor. For composite analyses, subscales were classified as adaptive (Positive Reframing, Emotional Support, Active Coping, Religion, Acceptance, Instrumental Support, Planning, Humor, Venting) or maladaptive (Self-Blame, Substance Use, Denial, Behavioral Disengagement, Self-Distraction), consistent with published factor structures. Internal consistency in the current sample ranged from acceptable to excellent across all subscales (Cronbach’s α: 0.612–0.902).

### 2.4. Statistical Analysis

All statistical analyses were performed using Python 3.12.3 (Python Software Foundation, Wilmington, DE, USA) with scipy (v1.17.1), scikit-learn (v1.8.0), pandas (v3.0.1), and numpy (v2.4.3). The significance threshold was set at α = 0.05 for all inferential tests. Non-normality of AUDIT scores was confirmed by Shapiro–Wilk testing (W = 0.727, *p* < 0.001), mandating non-parametric approaches for group comparisons throughout.

Mann–Whitney U tests (effect size: rank-biserial correlation r) compared AUDIT and coping scores between (a) males and females and (b) low-risk (AUDIT < 8) and at-risk drinkers (AUDIT ≥ 8). Effect sizes were interpreted as small (r = 0.10–0.29), medium (r = 0.30–0.49), and large (r ≥ 0.50). Spearman rank correlations characterized associations between AUDIT scores and all coping subscales. Multiple linear regression entered coping subscales with significant bivariate correlations alongside age and sex as covariates, using AUDIT total as the continuous outcome. Binary logistic regression employed at-risk status (AUDIT ≥ 8 vs. <8) as the dichotomous outcome; Wald statistics were computed as (B/SE)^2^ with chi-square(1) *p*-values; model performance was characterized by accuracy, sensitivity, specificity, and McFadden’s R^2^. Religion, which showed no independent contribution in the linear model (B = −0.017, *p* = 0.898), was excluded from the logistic model to maintain a parsimonious predictor set relative to the at-risk group size (*n* = 48), consistent with the recommendation of approximately 5–10 events per predictor variable.

For k-means cluster analysis, all 14 Brief COPE subscale scores were z-standardized prior to clustering. The optimal number of clusters was determined through a systematic evaluation of solutions ranging from k = 2 to k = 8. Three complementary criteria were applied: (1) within-cluster sum of squares (WCSS), plotted as a function of k, which showed a visible inflection at k = 5 (elbow method); (2) silhouette coefficients, which were low across all solutions (range: 0.07–0.12), consistent with the dimensional nature of coping data, but did not favor a competing solution over k = 5; and (3) substantive interpretability, assessed by examining the coping profiles produced at each k value. Solutions with fewer clusters (k = 3, k = 4) failed to separate the Substance-Oriented profile from other maladaptive configurations, while solutions with more clusters (k = 6, k = 7) produced progressively smaller and less interpretable subgroups without meaningful gains in separation. The five-cluster solution was selected as the most parsimonious configuration that preserved a clinically distinct substance-oriented coping profile while maintaining interpretable adaptive coping subtypes. Cluster stability was verified through 100-iteration bootstrapping with adjusted Rand index (ARI). Between-cluster AUDIT differences were tested using Kruskal–Wallis H statistics with Bonferroni-corrected Mann–Whitney U post hoc comparisons.

### 2.5. Ethical Considerations

Participation was entirely voluntary with no incentives offered. All data were anonymized prior to analysis. The protocol conformed to national and institutional regulations for observational studies involving human subjects.

## 3. Results

### 3.1. Sample Characteristics and Alcohol Use Patterns

The analytical sample comprised 244 medical students (mean age = 21.95 ± 3.27 years; range: 19–41), of whom 67.2% were female (*n* = 164) and 32.8% male (*n* = 80). Descriptive statistics for AUDIT scores and coping strategy composites are presented in Table 1.

Alcohol use was low overall (M = 4.88, SD = 5.80, median = 3), with marked right skewness (skewness = 2.39, kurtosis = 6.56) indicating that the distribution is driven by a minority of heavier consumers. As shown in Table 2, 19.7% of the sample (*n* = 48) exhibited at-risk drinking (AUDIT ≥ 8), including hazardous use (13.1%, *n* = 32), harmful use (3.3%, *n* = 8), and probable dependence (3.3%, *n* = 8). The majority remained in the low-risk category (80.3%, *n* = 196).

**Figure 1:**
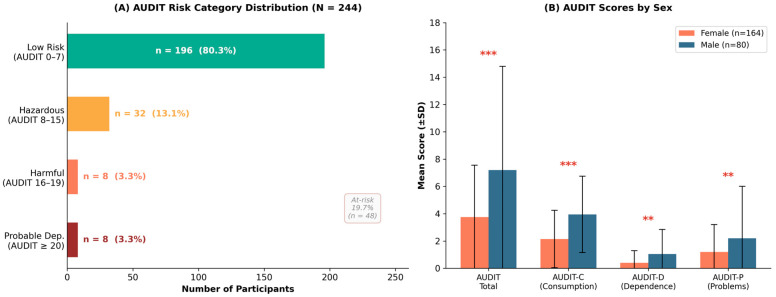
Alcohol use patterns in the study sample (*N* = 244). (**A**) Distribution of AUDIT risk categories according to WHO scoring guidelines. Bar colors encode the AUDIT risk severity gradient, progressing from green (Low Risk, AUDIT 0–7) through amber (Hazardous, AUDIT 8–15) and orange-red (Harmful, AUDIT 16–19) to dark red (Probable Dependence, AUDIT ≥ 20), with darker/warmer tones indicating increasing alcohol-related risk. The dotted line separates low-risk drinkers (AUDIT 0–7; 80.3%) from at-risk drinkers (AUDIT ≥ 8; 19.7%, *n* = 48), comprising hazardous, harmful, and probable dependence categories. (**B**) Mean AUDIT total and subscale scores by sex (±SD); female participants (*n* = 164) shown in coral, male participants (*n* = 80) in blue. Male students reported significantly higher scores across all four measures, with the largest effect observed for the Consumption subscale (r = −0.408). Mann–Whitney U tests; ** *p* < 0.01; *** *p* < 0.001.

Participants reported substantially higher adaptive coping (M = 55.91, SD = 7.26) than maladaptive coping (M = 21.54, SD = 5.28). The most endorsed strategies were Acceptance (M = 7.13), Planning (M = 6.99), Positive Reframing (M = 6.71), and Humor (M = 6.71). The least endorsed were Substance Use (M = 3.12) and Denial (M = 3.44)—a group-level picture whose apparent adaptive orientation, as the cluster analysis would reveal, conceals marked heterogeneity (Figure 1).

### 3.2. Sex Differences in Alcohol Use and Coping

Mann–Whitney U tests revealed significant sex differences across all four AUDIT measures (Table 3). Male students reported substantially higher total AUDIT scores (M = 7.20, SD = 7.58) compared to female students (M = 3.75, SD = 4.28; U = 8784, *p* < 0.001, r = −0.339), a medium effect. The sex gap was largest for the Consumption subscale (r = −0.408) and smallest for Dependence (r = −0.194), suggesting that male students drink more frequently and in larger quantities rather than displaying greater physiological dependence at this age.

**Figure 2:**
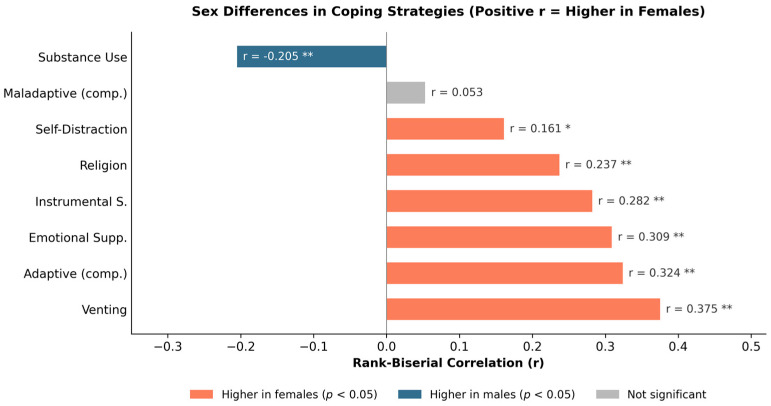
Sex differences in coping strategies expressed as rank-biserial correlations (r) from Mann–Whitney U tests (*N* = 244; female = 164, male = 80). Positive values indicate higher scores in female students; negative values indicate higher scores in males. Female students relied significantly more on social and support-oriented strategies (venting, emotional and instrumental support, religion), while male students reported higher substance use as a coping mechanism. Notably, no significant sex difference emerged for total maladaptive coping (r = 0.053, *p* = 0.502), indicating that the divergence in drinking risk is channeled specifically through substance use rather than reflecting globally elevated maladaptation in males. * *p* < 0.05; ** *p* < 0.01.

In the coping domain, female students relied significantly more on social and support-oriented strategies: emotional support (r = +0.309, *p* < 0.001), venting (r = +0.375, *p* < 0.001), instrumental support (r = +0.282, *p* < 0.001), and religion (r = +0.237, *p* = 0.002) were all higher in women. Total adaptive coping was significantly elevated in females (M = 57.26 vs. 53.15; r = +0.324, *p* < 0.001). Male students reported higher substance use as coping (r = −0.205, *p* = 0.004). Additionally, self-distraction was significantly higher in females (r = +0.161, *p* = 0.036). Importantly, no significant sex difference emerged for total maladaptive coping (*p* = 0.502), indicating that the sex divergence in drinking risk is not attributable to globally elevated maladaptation in males, it is channeled specifically through substance use (Figure 2).

### 3.3. Coping Profiles of At-Risk Versus Low-Risk Drinkers

Comparisons between low-risk (AUDIT < 8; *n* = 196) and at-risk drinkers (AUDIT ≥ 8; *n* = 48) revealed a striking and internally coherent pattern (Table 4). The largest effect was for substance use coping (M = 4.77 vs. 2.72; r = −0.597, *p* < 0.001), a large effect that captures the central argument of the study: substance use coping is not merely associated with drinking; it constitutes the dominant behavioral signature of the at-risk student.

Beyond substance use, at-risk students showed significantly higher behavioral disengagement (r = −0.294, *p* = 0.001) and maladaptive coping overall (M = 24.38 vs. 20.85; r = −0.338, *p* < 0.001). Notably, humor was also significantly elevated in the at-risk group (r = −0.289, *p* = 0.001), a finding that warrants careful interpretation and that distinguishes this reanalysis from conventional adaptive–maladaptive dichotomies. The protective side of the comparison confirmed planning (r = +0.321, *p* < 0.001), active coping (r = +0.190, *p* = 0.035), religion (r = +0.246, *p* = 0.007), instrumental support (r = +0.202, *p* = 0.027), and adaptive coping composite (r = +0.232, *p* = 0.013) as significant. At-risk students were also modestly younger (M = 21.04 vs. 22.17; *p* = 0.012), consistent with the known vulnerability of early academic exposure.

### 3.4. Correlations Between Alcohol Use and Coping Strategies

**Figure 3:**
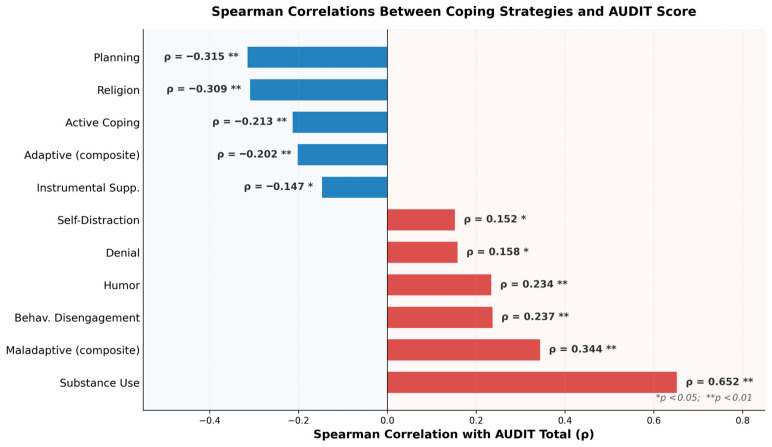
Spearman rank correlations (ρ) between Brief COPE subscales and AUDIT total score (*N* = 244). Bars are color-coded by direction of association: red bars indicate positive correlations (coping strategies associated with higher alcohol use, i.e., risk-associated), while blue bars indicate negative correlations (coping strategies associated with lower alcohol use, i.e., protective associations). Substance use coping dominated all other correlates (ρ = 0.652), followed by the maladaptive coping composite (ρ = 0.344). Among protective factors, planning (ρ = −0.315) and religion (ρ = −0.309) showed the strongest inverse associations. Humor, conventionally classified as an adaptive strategy, emerged as a positive correlate of AUDIT scores (ρ = 0.234), a finding that remained robust across all subsequent regression and cluster analyses. All displayed correlations reached statistical significance (* *p* < 0.05; ** *p* < 0.01).

Spearman correlations between AUDIT total and all Brief COPE subscales confirmed substance use coping as the dominant correlate (ρ = 0.652, *p* < 0.001), consistent with the group comparison data. Protective associations were observed for planning (ρ = −0.315, *p* < 0.001), religion (ρ = −0.309, *p* < 0.001), active coping (ρ = −0.213, *p* = 0.001), instrumental support (ρ = −0.147, *p* = 0.021), and adaptive coping composite (ρ = −0.202, *p* = 0.002). Positive correlations with AUDIT were found for behavioral disengagement (ρ = 0.237, *p* < 0.001), humor (ρ = 0.234, *p* < 0.001), denial (ρ = 0.158, *p* = 0.013), self-distraction (ρ = 0.152, *p* = 0.018), and maladaptive coping composite (ρ = 0.344, *p* < 0.001). The positive association of humor with AUDIT scores, not previously emphasized in this population, proved robust across all subsequent analyses (Figure 3).

### 3.5. Predictors of Alcohol Use: Multiple Linear Regression

Multiple linear regression (Table 5) confirmed substance use coping as the dominant independent predictor of AUDIT scores (B = 2.090, SE = 0.197; t = 10.61, *p* < 0.001; 95% CI [1.70, 2.48]), within a model explaining 51.3% of the variance in alcohol use (R^2^ = 0.513, Adjusted R^2^ = 0.493; F(10, 233) = 24.59, *p* < 0.001). Each unit increase in substance use coping was associated with a 2.09-point increase in AUDIT score, a relationship that survived adjustment for all other coping strategies, age, and sex. This proportion of explained variance should be interpreted with caution, as the Brief COPE Substance Use subscale and the AUDIT both assess self-reported alcohol-related behavior from different conceptual angles (coping function vs. consumption and consequences), and the overlap in item content likely inflates the apparent predictive power of substance use coping relative to psychologically independent predictors.

Male sex was the second most powerful predictor (B = 1.945, SE = 0.599; t = 3.25, *p* = 0.001; 95% CI [0.77, 3.12]), indicating that male students scored approximately 1.95 points higher on the AUDIT than females, independent of coping style. Planning was a significant protective predictor (B = −0.657, SE = 0.231; t = −2.84, *p* = 0.005; 95% CI [−1.11, −0.20]). A novel finding in this reanalysis was the emergence of humor as an independent positive predictor (B = 0.424, SE = 0.189; t = 2.24, *p* = 0.026; 95% CI [0.05, 0.80]) and self-distraction showed a marginally significant protective association (B = −0.342, *p* = 0.048; 95% CI [−0.68, −0.003]) that should be interpreted with caution given that the confidence interval barely excludes zero. Denial, behavioral disengagement, active coping, religion, and age did not contribute independently after full adjustment.

### 3.6. Logistic Regression: Predicting At-Risk Drinking Status

Binary logistic regression confirmed and extended the linear model’s findings (Table 6). Model performance: overall accuracy = 85.7%, specificity = 94.4%, sensitivity = 50.0%, McFadden R^2^ = 0.343. The moderate sensitivity reflects the inherent difficulty of classifying a minority outcome (19.7% at-risk) without sacrificing specificity.

**Figure 4:**
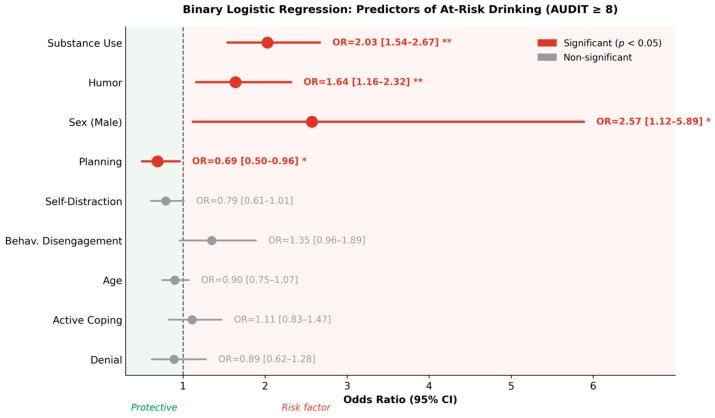
Forest plot of binary logistic regression predicting at-risk drinking status (AUDIT ≥ 8; *n* = 48) versus low-risk drinking (AUDIT < 8; *n* = 196). Red circles and lines indicate significant predictors (*p* < 0.05); grey indicates non-significant predictors. The dashed vertical line represents OR = 1 (null effect); values to the left indicate protective associations and values to the right indicate risk factors. Substance use coping was the strongest predictor (OR = 2.03), followed by humor (OR = 1.64) and male sex (OR = 2.57). Planning was the only significant protective factor (OR = 0.69). Statistical significance is indicated by asterisks: * *p* < 0.05; ** *p* < 0.01. Model performance: accuracy = 85.7%, sensitivity = 50.0%, specificity = 94.4%, McFadden R^2^ = 0.343.

Substance use coping significantly increased the odds of at-risk classification (OR = 2.026, 95% CI [1.54, 2.67]; Wald = 24.85, *p* < 0.001): each unit increase in this subscale was associated with a 103% increase in odds of at-risk classification. Humor emerged as the second-strongest independent predictor (OR = 1.638, 95% CI [1.16, 2.32]; Wald = 7.76, *p* = 0.005): each additional unit of humor coping was associated with a 64% increase in at-risk odds, a finding not previously reported in this population. Male sex was associated with a 2.6-fold elevation in odds (OR = 2.572, 95% CI [1.12, 5.89]; Wald = 4.99, *p* = 0.025), independently of all coping predictors. Planning remained protective (OR = 0.691, 95% CI [0.50, 0.96]; Wald = 4.76, *p* = 0.029): each unit increase in planning was associated with a 31% reduction in odds. No other coping variable reached significance, though behavioral disengagement approached the threshold (*p* = 0.080) and self-distraction showed a trend toward protection (*p* = 0.063) (Figure 4).

### 3.7. Coping Strategy Profiles: Cluster Analysis

K-means cluster analysis (k = 5) identified exploratory coping configurations (Table 7; Kruskal–Wallis H = 47.26, *p* < 0.001 for between-cluster AUDIT differences; silhouette coefficient = 0.094; bootstrap ARI = 0.488 ± 0.123 across 100 iterations) with markedly different alcohol use profiles.

Cluster 1—Isolated-Passive Copers (*n* = 7, 2.9%): The smallest cluster, defined by markedly low social support-seeking across all dimensions (Emotional Support M = 2.14, Instrumental Support M = 2.14, Venting M = 3.00), low active coping (M = 3.43), and low planning (M = 3.29). This profile is suggestive of social withdrawal and psychological isolation. AUDIT scores were low (M = 3.00 ± 3.61); the small sample size warrants interpretive caution.

Cluster 2—Emotionally Engaged Copers (*n* = 61, 25.0%): Defined by elevated emotional support, instrumental support, venting, and self-distraction alongside high self-blame (M = 6.82). This group was characterized by high relational and emotional investment in coping—directing distress outward, though not always adaptively. AUDIT scores were low (M = 3.21 ± 4.39).

Cluster 3—Balanced-Active Copers (*n* = 73, 29.9%): A broad adaptive profile combining the highest active coping (M = 7.34), planning (M = 7.62), acceptance (M = 7.64), positive reframing (M = 7.60), and humor (M = 7.19) in the sample. This cluster resembles a well-rounded problem-solving orientation. AUDIT scores were low (M = 3.78 ± 3.50).

Cluster 4—Detached-Cognitive Copers (*n* = 71, 29.1%): The second-largest cluster. Characterized by moderate problem-focused coping (Planning M = 7.06, Active Coping M = 6.04) combined with low emotional expressiveness and low social support-seeking (Venting M = 4.68, Emotional Support M = 4.07). This profile describes students who cope cognitively but maintain emotional distance. AUDIT scores were low-to-moderate (M = 4.13 ± 4.54).

Cluster 5—Substance-Oriented Copers (*n* = 32, 13.1%): The highest-risk coping profile. Defined by strikingly elevated substance use coping (M = 6.09; sample mean = 3.12), high behavioral disengagement (M = 5.16), high denial (M = 4.81), and high self-distraction (M = 7.00). This cluster reported AUDIT scores more than three times higher than the next-highest cluster (M = 12.66 ± 8.71), with the mean falling squarely within hazardous drinking territory. One in eight students, 13.1% of this cohort, belongs to this coping profile.

**Figure 5:**
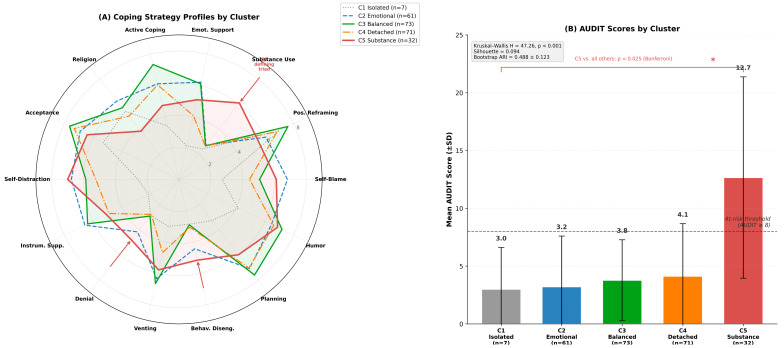
Coping strategy profiles and alcohol use across five k-means clusters (*N* = 244). (**A**) Radar plot of mean Brief COPE subscale scores for each cluster. The Substance-Oriented cluster (C5, red) is distinguished by markedly elevated substance use coping (M = 6.09 vs. sample mean 3.12), high behavioral disengagement, and high denial, forming an avoidance-based triad. The Balanced-Active (C3) and Emotionally Engaged (C2) clusters display broad adaptive profiles but differ in their reliance on cognitive versus social coping resources. (**B**) Mean AUDIT scores (±SD) by cluster; numeric values displayed above each bar represent the group mean AUDIT score. The Substance-Oriented cluster reported AUDIT scores more than three times higher than any other cluster (M = 12.66), with the mean falling within hazardous drinking territory. The dashed line indicates the at-risk threshold (AUDIT ≥ 8). Bonferroni-corrected post hoc Mann–Whitney U comparisons confirmed that C5 differed significantly from all other clusters (all *p* < 0.025), while no significant differences emerged among C1–C4 (all *p* > 0.15). Kruskal–Wallis H = 47.26, *p* < 0.001; silhouette coefficient = 0.094; bootstrap ARI = 0.488 ± 0.123 (100 iterations). Asterisks denote Bonferroni-corrected significance levels: * *p* < 0.05.

Bonferroni-corrected post hoc comparisons confirmed that the Substance-Oriented cluster differed significantly from all four other clusters (all *p* < 0.025), while no significant differences emerged among clusters 1–4 (all *p* > 0.15). This pattern suggests that alcohol risk may not be uniformly distributed but appears concentrated within an identifiable coping configuration. However, the low silhouette coefficient (0.094) and moderate bootstrap stability (ARI = 0.488 ± 0.123) indicate substantial overlap between clusters. These profiles should be understood as prototypical configurations representing regions of relatively higher density in a continuous coping space, not as discrete diagnostic categories, and individual assignments should be treated as probabilistic rather than definitive (Figure 5).

### 3.8. Summary of Key Findings

Across six complementary analyses, a consistent picture emerged. First, substance use coping is the primary behavioral mechanism predicting alcohol risk, with a correlation of 0.652, a regression coefficient explaining the majority of model variance, and a between-group effect size of r = 0.597. Second, male sex is a robust, independent structural risk factor, conferring a 2.6-fold elevation in at-risk odds. Third, planning is a modifiable coping factor consistently protective across both regression models. Fourth, humor, traditionally classified as an adaptive coping strategy, emerged as an independent risk factor for at-risk drinking across all analyses. Fifth, and most clinically relevant, alcohol risk appears concentrated within an identifiable coping profile, the Substance-Oriented Coper, comprising 13.1% of students, characterizable by their coping configuration, and in principle targetable before risk reaches clinical thresholds.

## 4. Discussion

### 4.1. Alcohol as Coping: Collapsing the Mediator and the Outcome

The dominant finding of this study is the strength of the association between substance use coping and AUDIT scores. This association was the largest in every analysis, bivariate correlations, group comparisons, linear regression, and logistic regression, and persisted after adjustment for all other coping strategies, age, and sex. The pattern is broadly consistent with self-medication and tension-reduction frameworks, which hold that alcohol is reinforced through its short-term anxiolytic properties. In a training environment that generates chronic stress while offering limited structured alternatives, the use of alcohol as a regulatory mechanism may become entrenched.

A methodological caveat is necessary. The Brief COPE Substance Use subscale (‘I’ve been using alcohol or other drugs to make myself feel better’; ‘I’ve been using alcohol or other drugs to help me get through it’) directly references the behavior that the AUDIT measures. The strong correlation between these instruments (ρ = 0.652) therefore partly reflects shared method variance: both capture self-reported substance use, albeit from different angles, one emphasizing coping function, the other consumption patterns and consequences. This overlap inflates the apparent predictive power of substance use coping relative to psychologically independent predictors such as planning or humor. The finding should therefore be interpreted not as evidence of a latent construct predicting drinking independently of drinking itself, but rather as evidence that students who cognitively frame their alcohol use as a coping tool report more consumption and more alcohol-related problems. The clinical implication, that interventions should target the coping function of alcohol, not just the stressor, remains relevant regardless of this methodological qualification.

More broadly, the cross-sectional nature of the data means that all observed associations, including the predictive models, are correlational. The regression terminology (‘predictor,’ ‘outcome’) reflects statistical convention rather than established causal direction. It is possible that students who already drink heavily retrospectively endorse substance use as a coping strategy, rather than coping style driving the initiation or escalation of drinking.

Programs that focus on reducing academic workload, improving scheduling, or providing emotional support will likely have limited reach among students for whom alcohol has already become the primary regulatory mechanism. This subgroup may require motivational interviewing, coping skills replacement training, and explicit discussion of the functional role alcohol plays in managing medical school-related stress.

### 4.2. Cluster Structure: Coping Profiles Rather than a Single Risk Continuum

The cluster analysis suggested that coping–alcohol relationships in this sample may be organized into distinguishable, though overlapping, configurations rather than along a single dimension of risk. The two most adaptive clusters (Emotionally Engaged and Balanced-Active) differed in their reliance on social versus cognitive coping, yet both maintained low AUDIT scores. This observation suggests that the specific content of adaptive coping may matter less than the absence of substance use coping as a primary strategy. The Isolated-Passive and Detached-Cognitive clusters represent intermediate profiles: students who do not rely on substances but also lack the social scaffolding observed in the more protected groups.

The Substance-Oriented cluster is the one with the clearest clinical relevance. Its defining features, elevated substance use coping, behavioral disengagement, and denial, form a combination that has been associated with the development of alcohol use disorders in prospective studies [21]. The fact that this cluster can be identified on the basis of coping profile alone, without requiring alcohol screening, raises the question of whether coping-based assessment could complement or even partially substitute for universal AUDIT screening in medical education settings.

Several limitations apply to the cluster solution. The silhouette coefficient (0.094) was low, indicating that the clusters do not form well-separated groups in the 14-dimensional coping space. This is a common finding in coping and personality research, where latent structure tends to be dimensional rather than categorical. The bootstrap ARI (0.488 ± 0.123) indicates that individual cluster assignments are only moderately stable across resampling, even if the overall pattern is interpretable. These clusters should therefore be understood as prototypical profiles with overlapping boundaries, not as discrete diagnostic categories. Future studies could consider latent profile analysis, which accommodates dimensional overlap and offers formal model comparison criteria (BIC, entropy) for determining the number of profiles.

### 4.3. Humor: An Adaptive Coping Strategy with Contextual Risk?

Among the less expected findings was the consistent association of humor with higher alcohol use risk. Humor is usually classified as adaptive, and meta-analytic evidence generally supports its protective effects on psychological adjustment. However, this classification treats humor as a unitary construct, collapsing several psychologically distinct forms [16].

In the context of alcohol use, one plausible interpretation is that humor functions as a social facilitator rather than a stress buffer [22]. Medical students who use humor to cope may also use humor to normalize the social contexts in which drinking occurs, post-exam gatherings, peer bonding events, and other situations where alcohol is the implicit social currency. The Brief COPE humor items (‘I make fun of the situation’; ‘I make jokes about it’) capture a strategy that is both cognitively adaptive and embedded in social settings where drinking and joking co-occur.

However, this interpretation is speculative given the cross-sectional design. The observed association could also reflect reverse causation, students who drink more may retrospectively endorse humor as a coping strategy because drinking contexts feel humorous, or confounding by personality traits such as sensation-seeking that independently predict both humor use and alcohol consumption.

From an intervention standpoint, the finding suggests caution in promoting “positive coping” without attention to contextual specificity. Programs that encourage humor-based coping without differentiating between its social subtypes may unintentionally reinforce drinking-associated behaviors. Future research should distinguish between affiliative/self-enhancing humor and humor employed in substance-associated social contexts, potentially using instruments such as the Humor Styles Questionnaire [23].

### 4.4. Sex Differences in Coping and Alcohol Use

Male sex was a significant independent predictor of at-risk drinking (OR = 2.572, *p* = 0.025), consistent with a large body of prior work [24]. More informative than the sex difference in drinking, however, is the specificity of the sex divergence in coping. Male students reported higher substance use coping, but there was no significant difference in overall maladaptive coping between men and women. Female students relied more heavily on social strategies, emotional support, instrumental support, venting, religion, while male students were more likely to manage distress through substance use. The observed pattern is consistent with a male-specific tendency to substitute chemical coping for social coping, a substitution with particular consequences in an environment that simultaneously rewards stoicism and provides easy access to alcohol.

This pattern likely reflects an interaction between professional culture and broader gender norms [25]. Medical training has traditionally emphasized self-sufficiency and emotional control, values that may be more compatible with drinking (socially acceptable, temporarily effective) than with help-seeking (sometimes perceived as weakness in competitive training environments). Alcohol prevention programming in medical schools may therefore benefit from explicitly validating and normalizing social support-seeking among male students, framing it as a professional skill rather than a sign of vulnerability.

### 4.5. Planning as a Modifiable Protective Factor

Planning was independently protective in both regression models, surviving adjustment for 13 other coping variables, age, and sex. This is noteworthy because planning, defined in the Brief COPE as deliberate step-sequencing and problem-structuring, is a teachable skill, not merely a personality trait [26].

Medical curricula already incorporate planning-related cognitive skills through clinical reasoning training and problem-based learning. The current findings suggest that extending these skills explicitly to personal stress management, through structured time management training, academic coaching, or goal-decomposition exercises, could yield protective effects on alcohol use alongside academic performance benefits. Framing alcohol prevention as a component of professional development, rather than as standalone addiction programming, may also improve institutional receptivity [27].

### 4.6. Implications for Student Mental Health Services

Taken together, the findings of this study point to several actionable implications for medical school wellness programs. First, coping-based screening using the Brief COPE, administered alongside or as a complement to the AUDIT, could help identify students whose drinking is embedded within a broader avoidance-oriented coping configuration, potentially enabling earlier and more targeted intervention. Second, planning skills training, including structured time management, goal decomposition, and problem-solving exercises, represents a low-stigma, curriculum-compatible intervention that may confer protective effects on both academic performance and alcohol use risk. Third, given the observed sex divergence in coping pathways, wellness programming could explicitly normalize social support-seeking among male students, framing it as a professional competency rather than a sign of vulnerability. Finally, the consistent association between humor and at-risk drinking suggests that peer-led social activities and student culture initiatives should be designed with awareness of the contexts in which humor and alcohol co-occur, rather than assuming that humor-based coping is uniformly protective.

### 4.7. Prevalence in Context

The 19.7% at-risk prevalence (AUDIT ≥ 8) is higher than the 10–15% typically reported in Western European medical student samples but consistent with Eastern European data more broadly. Romania’s per-capita alcohol consumption ranks among the highest in Europe, and social normalization of drinking in celebratory and social contexts may partially account for this. The figure also reflects the use of standard WHO AUDIT scoring, including the 0/2/4 scoring of items 9–10, which is more sensitive to alcohol-related consequences than compressed scoring alternatives sometimes used in other studies [28,29].

The prevalence underscores the practical relevance of the study findings: nearly one in five students meets at-risk criteria, and within this group, the Substance-Oriented coping profile represents a concentrated clinical need [30].

### 4.8. Strengths, Limitations, and Future Directions

This study has several methodological strengths: a sample size adequate for multivariate analyses (*N* = 244), integration of variable-centered and person-centered approaches, comprehensive coping assessment through a validated instrument, and a setting in an underrepresented Eastern European population. All analyses were conducted with open-source software (Python/scipy/scikit-learn), enabling computational reproducibility. The reanalysis from raw data corrected several statistical errors in an earlier version of this manuscript, including misspecified Wald statistics and *p*-value transpositions in the logistic model.

Limitations should also be acknowledged. The cross-sectional design does not permit causal inferences; it remains equally plausible that substance use coping leads to increased alcohol consumption, that established drinking patterns shape how students cognitively frame their coping repertoire, or that both are jointly determined by unmeasured third variables such as personality traits (e.g., sensation-seeking, impulsivity) or prior drinking history. Longitudinal and experimental designs are needed to disentangle these possibilities. Convenience sampling via an institutional platform likely introduced self-selection bias, and students with the most severe alcohol problems may be underrepresented, which would attenuate reported associations. The 67.2% female composition exceeds the typical sex ratio in Romanian medical faculties, suggesting differential participation rates. Self-report instruments are subject to social desirability bias. In particular, students may underreport alcohol consumption or overreport socially desirable coping strategies, a tendency that may be amplified in a medical student sample where professional identity is closely linked to health-promoting behavior. Furthermore, this study was conducted at a single institution (University of Medicine and Pharmacy of Craiova), which limits generalizability to other Romanian medical faculties or to medical student populations in different cultural and educational contexts. Multi-center studies including institutions with different admission structures, curricular designs, and student support services would be needed to determine whether the observed coping profiles and their association with alcohol use replicate beyond this setting. The Isolated-Passive cluster (*n* = 7) is too small for reliable characterization. The Spearman correlations in Section 3.4 were not corrected for multiple comparisons; the weakest associations (denial, ρ = 0.158; self-distraction, ρ = 0.152) would not survive Bonferroni correction and should be considered exploratory.

Future research should (1) use longitudinal designs tracking students across pre-clinical and clinical years to examine profile stability; (2) test the mediating role of burnout and depression in the coping–alcohol relationship [23]; (3) develop brief coping-based screening tools suitable for medical school wellness programs; (4) evaluate planning-skills training in randomized trials as a component of alcohol prevention; and (5) examine humor subtypes as differential predictors, using instruments that distinguish affiliative from aggressive and self-defeating humor [15,31].

## 5. Conclusions

This study examined alcohol use in medical students through the lens of coping behavior rather than stress exposure alone. The data indicate that for a subgroup of students, comprising approximately 13% of this cohort, alcohol use is organized as a primary coping strategy within a broader profile of avoidance-oriented behavior (substance use coping, behavioral disengagement, denial). Substance use coping was the strongest predictor of AUDIT scores across all analytical approaches, with a bivariate correlation of ρ = 0.652, a between-group effect size of r = 0.597, and a regression model accounting for over half of the variance in alcohol use (R^2^ = 0.513). Although this coping profile is statistically distinguishable from the remaining clusters, the dimensional nature of coping data means that boundaries between profiles are gradual rather than categorical, and future research using latent profile analysis may better accommodate this overlap.

Several practical implications follow. First, universal stress-reduction programs are unlikely to reach the Substance-Oriented subgroup, which requires targeted intervention addressing the coping function of alcohol, though the logistic model’s sensitivity of 50% indicates that coping-based screening alone would miss approximately half of at-risk students and would need further development before clinical application. Second, planning skills training has potential as a dual-purpose intervention targeting both academic effectiveness and alcohol risk. Third, the finding that male students substitute social coping with substance use coping suggests that sex-specific programming, explicitly normalizing help-seeking in male students, may address an upstream vulnerability. Fourth, humor-based coping, conventionally classified as adaptive, was associated with increased alcohol risk in this population. This warrants further investigation with instruments that differentiate humor subtypes before it can be incorporated into intervention design.

The coping patterns that medical students develop during their training are likely to persist into professional practice. Understanding these patterns and intervening where they are maladaptive is relevant not only for student wellbeing but, by extension, for the quality of care they will eventually provide.

## Figures and Tables

**Table 1:** Descriptive Statistics for AUDIT Scores and Coping Composites (*N* = 244).

Variable	M	SD	Median	Range	Skew	Kurt
AUDIT Total	4.88	5.80	3.0	0–32	2.39	6.56
AUDIT-C (Consumption)	2.76	2.43	2.0	0–12	1.27	1.54
AUDIT-D (Dependence)	0.62	1.60	0.0	0–12	3.99	19.42
AUDIT-P (Problems)	1.50	2.81	0.0	0–16	2.71	8.37
Adaptive Coping	55.91	7.26	57.0	22–70	−1.01	2.04
Maladaptive Coping	21.54	5.28	21.0	10–40	0.34	0.19

Note. M = mean; SD = standard deviation; Skew = skewness; Kurt = kurtosis. AUDIT-C = Consumption subscale; AUDIT-D = Dependence symptoms; AUDIT-P = Alcohol-related problems. Cronbach’s α values ranged from 0.612 (Emotional Support) to 0.902 (Religion).

**Table 2:** Distribution of AUDIT Risk Categories (*N* = 244).

Risk Category	AUDIT Range	*n*	%
Low Risk	0–7	196	80.3%
Hazardous	8–15	32	13.1%
Harmful	16–19	8	3.3%
Probable Dependence	20–40	8	3.3%

Note. AUDIT = Alcohol Use Disorders Identification Test. Risk categories applied per WHO AUDIT scoring guidelines. At-risk = AUDIT ≥ 8 (*n* = 48, 19.7%).

**Table 3:** Sex Differences in Alcohol Use and Coping Strategies (*N* = 244).

Variable	Female M (SD)	Male M (SD)	U	*p*	r
AUDIT Total	3.75 (4.28)	7.20 (7.58)	8784	<0.001	−0.339
AUDIT-C	2.18 (1.99)	3.95 (2.79)	9240	<0.001	−0.408
AUDIT-D	0.40 (1.12)	1.06 (2.23)	7833	0.001	−0.194
AUDIT-P	1.17 (2.18)	2.19 (3.71)	7959	0.003	−0.213
Substance Use	2.96 (1.53)	3.46 (1.70)	7903	0.004	−0.205
Emotional Supp.	5.56 (1.51)	4.75 (1.49)	4530	<0.001	+0.309
Instrumental S.	5.98 (1.51)	5.12 (1.69)	4713	<0.001	+0.282
Religion	5.68 (2.06)	4.81 (2.12)	5002	0.002	+0.237
Self-Distraction	6.13 (1.71)	5.60 (1.84)	5502	0.036	+0.161
Venting	6.09 (1.48)	5.16 (1.36)	4102	<0.001	+0.375
Adaptive Coping	57.26 (6.61)	53.15 (7.78)	4436	<0.001	+0.324
Maladaptive	21.73 (5.13)	21.15 (5.59)	6213	0.502	+0.053

Note. M = mean; SD = standard deviation; U = Mann–Whitney U statistic; *p* = *p*-value; r = rank-biserial correlation. AUDIT-C = Consumption subscale; AUDIT-D = Dependence symptoms; AUDIT-P = Alcohol-related problems; Supp. = Support. Negative r indicates higher scores in males. Only variables with *p* < 0.05 shown for coping subscales; non-significant coping subscales omitted for clarity.

**Table 4:** Coping Strategy Differences Between Low-Risk and At-Risk Drinkers.

Variable	Low-Risk M (SD)	At-Risk M (SD)	U	*p*	r
Substance Use	2.72 (1.16)	4.77 (2.04)	7510	<0.001	−0.597
Behav. Disengage.	3.46 (1.32)	4.29 (1.66)	6086	0.001	−0.294
Humor	6.57 (1.53)	7.27 (1.30)	6062	0.001	−0.289
Maladaptive Coping	20.85 (4.66)	24.38 (6.63)	6294	<0.001	−0.338
Planning	7.14 (1.20)	6.38 (1.47)	3196	<0.001	+0.321
Religion	5.59 (2.04)	4.62 (2.28)	3548	0.007	+0.246
Active Coping	6.30 (1.66)	5.79 (1.71)	3810	0.035	+0.190
Instrumental Supp.	5.82 (1.59)	5.23 (1.68)	3752	0.027	+0.202
Adaptive Coping	56.53 (6.86)	53.40 (8.33)	3614	0.013	+0.232
Age	22.17 (3.45)	21.04 (2.21)	3632	0.012	+0.228

Note. M = mean; SD = standard deviation; U = Mann–Whitney U statistic; *p* = *p*-value; r = rank-biserial correlation. Low-risk = AUDIT < 8 (*n* = 196); At-risk = AUDIT ≥ 8 (*n* = 48). Negative r indicates higher scores in at-risk group; positive r indicates higher scores in low-risk group.

**Table 5:** Multiple Linear Regression Predicting AUDIT Total Score (*N* = 244).

Predictor	B	SE	t	*p*	95% CI
Substance Use	2.090	0.197	10.61	<0.001	[1.70, 2.48]
Sex (Male)	1.945	0.599	3.25	0.001	[0.77, 3.12]
Planning	−0.657	0.231	−2.84	0.005	[−1.11, −0.20]
Humor	0.424	0.189	2.24	0.026	[0.05, 0.80]
Self-Distraction	−0.342	0.172	−1.98	0.048	[−0.68, −0.00]
Denial	0.292	0.233	1.26	0.210	[−0.17, 0.75]
Behav. Disengage.	0.196	0.226	0.87	0.387	[−0.25, 0.64]
Active Coping	0.144	0.177	0.81	0.418	[−0.21, 0.49]
Religion	−0.017	0.134	−0.13	0.898	[−0.28, 0.25]
Age	−0.082	0.084	−0.99	0.325	[−0.25, 0.08]

Note. B = unstandardized regression coefficient; SE = standard error; t = t-statistic; *p* = *p*-value; CI = confidence interval. Significant predictors (*p* < 0.05), R^2^ = 0.513, Adjusted R^2^ = 0.493; F(10, 233) = 24.59, *p* < 0.001.

**Table 6:** Binary Logistic Regression Predicting At-Risk Drinking Status (AUDIT ≥ 8).

Predictor	B	SE	Wald	*p*	OR	95% CI
Substance Use	0.706	0.142	24.85	<0.001	2.026	[1.54, 2.67]
Humor	0.494	0.177	7.76	0.005	1.638	[1.16, 2.32]
Sex (Male)	0.945	0.423	4.99	0.025	2.572	[1.12, 5.89]
Planning	−0.369	0.169	4.76	0.029	0.691	[0.50, 0.96]
Self-Distraction	−0.241	0.130	3.46	0.063	0.785	[0.61, 1.01]
Behav. Disengage.	0.301	0.172	3.07	0.080	1.351	[0.96, 1.89]
Age	−0.108	0.092	1.40	0.236	0.897	[0.75, 1.07]
Active Coping	0.101	0.144	0.49	0.484	1.106	[0.83, 1.47]
Denial	−0.119	0.186	0.41	0.524	0.888	[0.62, 1.28]

Note. B = unstandardized regression coefficient; SE = standard error; OR = odds ratio; CI = confidence interval; Wald = Wald chi-square statistic. Significant predictors (*p* < 0.05), Accuracy = 85.7%; Sensitivity = 50.0%; Specificity = 94.4%; McFadden R^2^ = 0.343. Predictors ordered by statistical significance.

**Table 7:** Coping Strategy Profiles and AUDIT Scores Across Five Clusters (k-means, k = 5; *N* = 244).

Coping Strategy	C1 Isolated (*n* = 7)	C2 Emotional (*n* = 61)	C3 Balanced (*n* = 73)	C4 Detached (*n* = 71)	C5 Substance (*n* = 32)
Self-Blame	2.71	6.82	5.07	4.44	6.12
Positive Reframing	7.00	6.07	7.60	6.85	5.53
Substance Use	2.43	2.72	2.68	2.65	6.09
Emotional Support	2.14	6.21	6.11	4.07	5.09
Active Coping	3.43	6.11	7.34	6.04	4.72
Religion	5.43	6.25	5.71	5.04	3.84
Acceptance	5.29	6.90	7.64	7.31	6.41
Self-Distraction	2.57	6.77	5.86	5.23	7.00
Instrumental Supp.	2.14	6.57	6.38	4.89	5.06
Denial	2.86	4.18	2.92	2.79	4.81
Venting	3.00	6.34	6.66	4.68	5.78
Behav. Disengage.	2.86	4.43	2.90	3.06	5.16
Planning	3.29	7.10	7.62	7.06	6.00
Humor	4.14	6.46	7.19	6.61	6.88
AUDIT (M ± SD)	3.00 ± 3.61	3.21 ± 4.39	3.78 ± 3.50	4.13 ± 4.54	12.66 ± 8.71

Note. AUDIT = Alcohol Use Disorders Identification Test; M = mean; SD = standard deviation. C1–C5 = Clusters 1–5. Cluster labels assigned by authors based on dominant coping characteristics. Kruskal–Wallis H = 47.26, *p* < 0.001. Silhouette = 0.094. Bootstrap ARI = 0.488 ± 0.123 (100 iterations).

## Data Availability

Data available upon request from one of the authors.

## Abbreviations

The following abbreviations are used in this manuscript:

AUDIT	Alcohol Use Disorders Identification Test
AUDIT-C	AUDIT Consumption subscale
AUDIT-D	AUDIT Dependence symptoms subscale
AUDIT-P	AUDIT Alcohol-related Problems subscale
ARI	Adjusted Rand Index
Brief COPE	Brief Coping Orientation to Problems Experienced
CI	Confidence Interval
OR	Odds Ratio
SD	Standard Deviation
SE	Standard Error
WHO	World Health Organization
